# Accurate statistics for local sequence alignment with position-dependent scoring by rare-event sampling

**DOI:** 10.1186/1471-2105-12-47

**Published:** 2011-02-03

**Authors:** Stefan Wolfsheimer, Inke Herms, Sven Rahmann, Alexander K Hartmann

**Affiliations:** 1 Laboratoire MAP5 (UMR CNRS 8145), Université Paris Descartes, 75006 Paris, France; 2 Technische Fakultät, Universität Bielefeld, 33501 Bielefeld, Germany; 3 Department of Computer Science, TU Dortmund, Germany; 4 Institut für Physik, Universität Oldenburg, D-26111 Oldenburg, Germany

## Abstract

**Background:**

Molecular database search tools need statistical models to assess the significance for the resulting hits. In the classical approach one asks the question how probable a certain score is observed by pure chance. Asymptotic theories for such questions are available for two random i.i.d. sequences. Some effort had been made to include effects of finite sequence lengths and to account for specific compositions of the sequences. In many applications, such as a large-scale database homology search for transmembrane proteins, these models are not the most appropriate ones. Search sensitivity and specificity benefit from position-dependent scoring schemes or use of Hidden Markov Models. Additional, one may wish to go beyond the assumption that the sequences are i.i.d. Despite their practical importance, the statistical properties of these settings have not been well investigated yet.

**Results:**

In this paper, we discuss an efficient and general method to compute the score distribution to any desired accuracy. The general approach may be applied to different sequence models and and various similarity measures that satisfy a few weak assumptions. We have access to the low-probability region ("tail") of the distribution where scores are larger than expected by pure chance and therefore relevant for practical applications. Our method uses recent ideas from rare-event simulations, combining Markov chain Monte Carlo simulations with importance sampling and generalized ensembles. We present results for the score statistics of fixed and random queries against random sequences. In a second step, we extend the approach to a model of transmembrane proteins, which can hardly be described as i.i.d. sequences. For this case, we compare the statistical properties of a fixed query model as well as a hidden Markov sequence model in connection with a position based scoring scheme against the classical approach.

**Conclusions:**

The results illustrate that the sensitivity and specificity strongly depend on the underlying scoring and sequence model. A specific ROC analysis for the case of transmembrane proteins supports our observation.

## Background

A large amount of molecular biological data is stored in form of sequences of symbols in huge data bases, e.g., DNA sequences or the primary structure of proteins. It is one main task of Bioinformatics [1] to develop algorithms [2] which allow to find, via sequence comparison, for a given "query" sequence the most similar "subject" sequences in a data base. All widely-used algorithms depend on many parameters, the so-called *scoring schemes *[2], which are usually suitably adopted to test sets of data. Parts of these scoring schemes involve also rules how to deal with small non-similar subsequences, the so-called *gaps*. The most popular sequence-comparison algorithms are the Smith-Waterman algorithm [3] for pairwise local sequence alignment (which means that the most similar subsequences of two sequences are found) and the Viterbi algorithm for sequence-to-HMM alignment. In the latter case, one specific sequence is compared to a full set of sequences which is specified via a Hidden Markov Model (HMM) [4]. For practical applications, often very fast heuristics are used, which do not find the exact best-matching (sub-)sequences but only approximations of the optimum. The BLAST algorithm [5] is used widely. Sequence-comparison algorithms return a *raw similarity score*, i.e., a number, that quantifies the similarity between the input objects. Unfortunately, this raw score is hard to interpret because one does not know its absolute scale.

An interpretation becomes possible when we specify a probabilistic null model for the input: Then the similarity score becomes a random variable *S *whose probabilities Prob(*S *= *s*) under the null model can be determined. Sometimes this can be done analytically, but usually one has to apply numerical simulation [6]. The *p-value *assigned to an observed score *s *is defined as *pval*(*s*): = Prob(*S *≥ *s*) in the null model, and log *pval*(*s*) is a measure of surprise (and hence a universally normalized score) for *s*. The key problem is, of course, to find Prob(*S *= *s*) for a given sequence-comparison algorithm, a given scoring scheme, and a given null model.

In this paper, we explain and extend an *efficient *and *generally applicable *numerical technique that solves this problem in many different sequence comparison settings, such as for a BLAST-like database search [5] with a fixed query, for position-specific scoring and/or gap-cost schemes (essentially HMMs), or for normalized alignment [7]. In each of those settings a variety of null models in addition to the i.i.d. model is possible.

### Previous work

We start by introducing some necessary formal notations. For a full description, please refer to Ref. [2]. Let Σ be a fixed alphabet of symbols, denoting e.g. nucleotides (|Σ| = 4) or amino acids (|Σ| = 20).

Most of the existing statistical work for pairwise sequence comparison focuses on null models where both sequences are random and at each position a symbol σ ∈ Σ is chosen independently of the other positions ("i.i.d. model"), with a given frequency $f_{\sigma }\;>\;0\;\left(\Sigma_{\sigma \in \Sigma }\;f_{\sigma }\;=\;1\right)$. *f *often reflects the average composition of proteins in the UniProt/SwissProt database [8]. An alignment of the two sequences is a set of *pairs *{(*i_k_*, *j_k_*)}, which means that symbol of position *i_k _*of the first sequence is aligned (or paired) to the symbol at position *j_k _*of the second sequence. The pairs must not cross, i.e., if for two pairs (*i_k_*, *j_k_*) (*i_l_*, *j_l_*) the condition *i_k _*<*i_l _*holds, then also *j_k _*<*j_l_*. Positions which are not paired, i.e., which do not appear in the alignment, contribute to the above mentioned gaps. The length of a gap is the number of adjacent ungapped positions. In the following example, where the two sequences are shown such that paired symbols are atop on each other,

$$\begin{array}{l} \begin{array}{ccccccccccccc} \text{Q} & \text{G} & \text{E} & \text{G} & \text{G} & \text{D} & \text{A} & - & - & - & \text{W} & \text{C} & \\ \text{Q} & \text{G} & - & - & \text{G} & \text{D} & \text{A} & \text{T} & \text{T} & \text{T} & \text{W} & \text{C} & \Leftrightarrow \end{array}\\ \left\{(1,1),(2,2),(5,3),(6,3),(7,5)\;(8,9),(9,10)\right\} \end{array}$$

two gaps of lengths two and three appear.

Scores for individual pairs of symbols are given by a constant (position-independent) symmetric Σ × Σ scoring matrix with negative expected score, such as BLOSUM62 [9]. The score of an alignment is given by the sum of the scores of the pairs, plus (negative) contributions for the gaps. For the conventional gap-scoring schemes (called "affined"), each gap contributes a score which depends only linearly on its length (times a parameter called *gap-extension penalty*) plus a constant (*gap-open penalty*). We shall refer to this model later as "random query - general-purpose scoring" (RQGS).

For gapless pairwise local sequence alignment, the raw score distribution can be derived numerically by Markov chain analysis [10] and also asymptotically for infinite sequences (Karlin-Altschul or Dembo-Karlin statistics [11]): It is an extreme-value distribution (EVD), also called Gumbel distribution [12]:

(1) $$\begin{array}{ccc} \text{Prob}(S>s) & = & 1-\exp[-c\cdot \exp(-\lambda s)]\\ & \Leftrightarrow & \\ \text{Prob}(S>s) & = & \begin{array}{l} c\cdot \lambda \cdot \exp(-\lambda s)\times \\ \exp[-c\cdot \exp(-\lambda s)] \end{array} \end{array}$$

where the parameters λ > 0 and *c *> 0 depend on the score matrix, on the symbol frequencies *f*, and on the query and subject sequence lengths *L*_Q _and *L*_S_. Asymptotically we have *c *= *KL*_Q_*L*_S _for a length-independent *K *> 0.

For gapped pairwise local sequence alignment, which is the most relevant case in database queries there exist no universal analytic results, with the exception of few special cases [13]. Empirical evidence also indicates convergence towards the Gumbel form for long sequences; λ and *K *additionally depend on the gap-cost function [14]. Several works have focused on efficient numerical estimation of these parameters [15]. The influence of varying lengths of the finite sequences [16] is treated in various ways, e.g. by adjusting the lengths of the sequences to "effective lengths" but still assuming a Gumbel form of the distribution. Nevertheless, for moderate sequence lengths, which are biologically most relevant, the true distribution differs strongly from a Gumbel form [17,18], which can be dealt with by including a correction term to the Gumbel form (giving rise to an additional parameter).

The (RQGS) model is convenient, because the problem of computing significance values reduces to the estimation of only two parameters, which can be precomputed for each scoring scheme. However, there are also several problems. For instance, even if one considers just the gapless case, it is in general not easy to extend the analytic asymptotic theory to more complex null models. Furthermore, for practical applications where finite sequence lengths are considered, of even more importance is: The p-values reported by (the original) BLAST only depend on the raw score, the query and the subject length, and not on the actual query sequence. This leads to large distortions when the composition of the query sequence does not match the composition of the null model. For example, when we run a homology search for the human transmembrane protein rhodopsin (UniProt accession P08100) with BLAST (BLOSUM 62, gap-init 12, gap-extend 1, no composition adjustment, no filtering), we find a possibly remote homolog Q8NH42 with an E-value of 9 · 10^-8^. The E-value for score *s *is the expected number of database hits with score at least *s *and can be easily computed from *pval*(*s*) and the database size. Hence, it appears unlikely to obtain such a homolog by pure chance, i.e., the homolog appears to be relevant. However, using a recent "composition-based adjustment" option [19,20] leads to a very different E-value of 0:001 for the same protein. This underlines the importance of query-specific or at least composition-based statistics, particularly for intermediate p-values.

The statistics of position-dependent scoring and/or gap-cost schemes, as used in PSI-BLAST [21] or in hidden Markov model (HMM) frameworks, are less well explored. The central question here is, "given a query Q and a position-specific scoring scheme, what is the score distribution when random null-model sequences of given length are scored against Q?". We refer to this model as "fixed query - position-dependent scoring" (FQPS). As a compromise between the general (RQGS) and the very specific (FQPS) models, one may neither use a completely free (i.i.d) nor use a fixed query but draw query sequences according to more specific models, e.g., HMMs for transmembrane proteins.

In all these cases EVDs Eq. (2) may still used heuristically by fitting the parameters of the EVD to the simulated data. This can be achieved by generating pairs of random sequences according to the given null model while recording the histogram of observed alignment scores. Using such a "simple sampling" approach, the large-probability region of the score distribution can be investigated, e.g., for probabilities about > 10^-4 ^when generating 10^4 ^sequence pairs. Such an approach is implemented, e.g. in the hmmcalibrate program from the HMMER package [22]. Nevertheless, this procedure may fail to describe distribution in the "rare-even tail", i.e., where the probability is small (say < 10^-4^), although this part of the distribution is most important for the estimation of the statistical significance.

**Figure 1:**
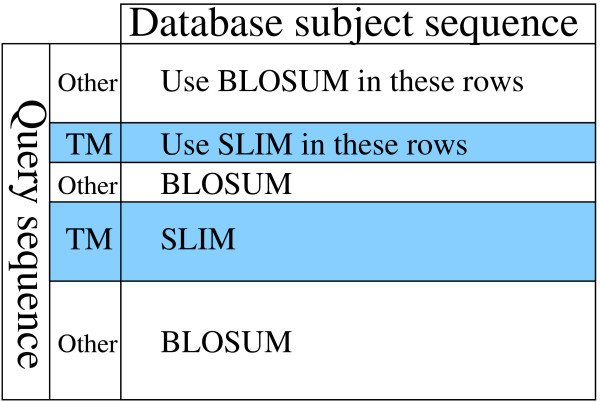
**Bipartite scoring scheme**. Bipartite scoring scheme for the detection of homologous transmembrane proteins from Ref. [23]. The figure represents the Smith-Waterman alignment matrix and indicates which scoring matrix is used for which query positions (rows): In transmembrane helices, a transmembrane-specific scoring matrix is used. For p-value computations, the query is assumed fixed or generated by the TMHMM and the subject is assumed a random i.i.d. sequence drawn from the distribution of amino-acid frequencies of the database.

Our motivation for a simulation-based method that makes no initial parametric assumption refers to the approach [23] to increase the sensitivity of detecting homologs of a given transmembrane (TM) protein in a database search: A bipartite scoring scheme with a (non-symmetric) transmembrane helix specific scoring matrix (such as SLIM [23]) for the TM helices and a general-purpose scoring matrix (such as BLOSUM [9]) for the remaining regions of the query protein were applied, see Figure 1. This results in higher search sensitivity and specificity. However, a statistical theory or efficient computational method to obtain the score probabilities in such a (FQPS) framework is missing so far.

### Our contributions and paper outline

We present a general framework for efficient estimation of raw score distributions in sequence comparison problems. In particular the rare-event tail for large scores can be accessed. We only make the following assumptions:

1. We are able to sample pairs *x*, *y *of sequences according to the null model and to compute the null model probability of any given *x*, *y*.

2. We have an efficient algorithm A that computes the score *S*(*x*, *y*), where *x*, *y *could be a pair of randomly drawn sequences (RQGS or HMM), or one fixed and one random sequence (FQPS).

3. The scores are rational numbers with a common denominator. Hence, without loss of generality, they can be assumed to be integers.

4. Optionally for the (HMM) approach, we have an efficient algorithm V that predicts the most likely state sequence for a given sequence.

Our framework is readily applicable to the (RQGS), (FQPS) and (HMM) models, but also to more exotic settings, such as normalized alignment [7], where the score is not additive, but normalized by the alignment length, for which no statistical framework exists so far. Very recently Eddy [24] studied the distributions of Viterbi and Forward scores under probabilistic local alignment, for which a numerical analysis of the rare-event tail would be of interest as well.

In the current stage of the methodology, the computation of an accurate "on the fly" p-value for each particular database query might be impracticable as each full calculation is not achieved within a few minutes.

We will illustrate the approach for the HMM for TM proteins (TMHMM [25,26]), which has been proven valuable in predicting TM helices. In this approach (and possible in other models as well) one is able to specify the score distribution in more detail. Each query may be classified to be a member of certain given sub-classes C. In this case, it could be meaningful to obtain an individual score distribution for each subclass. A natural classification of the TMHMM is the number of transmembrane regions. Since for a given sequence this number is usually not known exactly, one takes the most likely one.

The rest of the paper is organized as follows. The following section presents the mathematical background on importance sampling and Markov chain Monte Carlo methods which are fundamental to the methods used to obtain the score distribution, in particular in the rare-event tail, for different null models. Next, we present a description of the methodology. Section "Results" shows computational results on transmembrane protein similarity statistics in (RQGS), (FQPS) and (HMM). A discussion closes the paper.

## Methods

### Importance sampling

Importance sampling is a general technique to reduce the variance in the estimation of quantities that can be written as an expectation E[h(Z)], where *Z *is a random object representing the null model and *h *is a real-valued function. We assume that we can draw within a computer simulation *n *random samples *z*_1_, ..., *z_n _*according the null model. The expectation is then approximated by the empirical mean $E[h(Z)]\approx 1/n\cdot \sum_{i=1}^{n}h(z_{i})$.

In our setting, to estimate the score distribution (and then p-values), we consider the state space $Z=\Sigma^{L_{\text{Q}}}\times \Sigma^{L_{\text{S}}}$, from which we generate *n *random pairs of sequences({*x*^(1) ^, *y*^(1)^} ,..., {*x*^(*n*) ^, *y*^(*n*)^}).

These pairs are then aligned by a given algorithm A and the corresponding similarity scores *S*(*x*^(*i*)^, *y*^(*i*)^) are computed. To formally write a histogram as an expectation value, we consider the family of indicator functions $h_{s}=Z\to \{0,1\}$ for all *s *> 0, defined by *h_s_*(*x*, *y*) := 1 if *S*(*x*, *y*) = *s*, and *h_s_*(*x*, *y*) := 0 if *S*(*x*, *y*) ≠ *s*, hence

$$\begin{array}{lll} \text{Prob}\;(S(X,Y)=s) & = & E[h_{s}(X,Y)]\\ & \approx & |\;\{i:S(x^{\left(i\right)},y^{\left(i\right)})=s\}|/n \end{array}$$

This means, we approximate the unknown exact probability Prob(*S*(*X, Y*)) by normalized score histograms over all sampled sequence pairs. If the probability to be estimated is small, say 10^-9^, when using simple sampling, we need about 10^12 ^samples to estimate it with reasonable precision. Thus, for very rare events, this sampling quickly becomes infeasible.

Importance sampling generates the "interesting" events more often by sampling from a different distribution and correcting for this bias afterward, which results in a more accurate estimate with a reasonable number of samples. Let *p *be the probability mass function (pmf) of (*X*, *Y*), and let *q *be another pmf satisfying *q*(*x*, *y*) > 0 whenever *p*(*x*, *y*) > 0. Then

(2) $$\begin{array}{c} E_{p}\left[h\left(X,Y\right)\right]=\sum_{x,y}h\left(x,y\right)\cdot p\left(x,y\right)\\ =\sum_{x,y}h\left(x,y\right)\cdot \frac{p\left(x,y\right)}{q\left(x,y\right)}\cdot q\left(x,y\right)\\ =E_{q}\left[h\left(X^{\prime },Y^{\prime }\right)\frac{p\left(X^{\prime },Y^{\prime }\right)}{q\left(X^{\prime },Y^{\prime }\right)}\right]\\ \approx \frac{1}{n}\sum_{i=1}^{n}h({x^{\prime }}^{\left(i\right)},{y^{\prime }}^{\left(i\right)})\cdot \frac{p({x^{\prime }}^{\left(i\right)},{y^{\prime }}^{\left(i\right)})}{q({x^{\prime }}^{\left(i\right)},{y^{\prime }}^{\left(i\right)})}, \end{array}$$

where each pair (*x'*^(*i*)^, *y'*^(*i*)^) is sampled from the pmf *q*. Eq. (2) gives us the relationship between the expectation value w.r.t. the unknown distribution of interest, the target distribution *p*, and samples drawn from the actually used sampling distribution *q*. To successfully apply importance sampling, *q *has to fulfill three properties: First, it needs to put high probability on the region of interest; second, we need to be able to sample according to *q*; third, we need to be able to compute the correcting weight *p*(*x*, *y*)/*q*(*x*, *y*). Since directly sampling from *q *often is impossible, we shall use a general sampling method which we describe next.

### Metropolis-Hastings sampling

If we need to generate samples from a discrete (or continuous) distribution *q *but have no simple direct method to do so, the Metropolis-Hastings method [27] provides a solution by constructing an ergodic Markov chain with stationary distribution *q *in the following way.

Extensive introductions to such so-called Monte Carlo simulations can be found, e.g., in Refs. [28,29]. Here we just give a concise introduction specifically tailored to the problem of sampling pairs of sequences. Let us call the elements (*x*, *y*) of the sample space Z*configurations*. The sampling will be performed by randomly "moving" in the space of configurations, such that for each step a configuration is altered only slightly. Configurations which can be visited and are connected by a move are called neighbors. Hence, each configuration (*x*, *y*) has a algorithm-dependent set N(x,y) of potential neighbors (*x'*, *y'*) ∈ N(x,y). The movement is performed in such a way that each neighbor exhibits a positive probability *P*_(*x*,*y*), (*x'*,*y'*) _as being the next configuration. The proposal is *accepted *with probability

(3) $$\begin{array}{ll} & \alpha \left(\left(x,y\right)\to \left(x^{\prime },y^{\prime }\right)\right)\\ = & \min\left\{1,\frac{q\left(x^{\prime },y^{\prime }\right)\cdot P_{\left(x^{\prime },y^{\prime }),(x,y\right)}}{q(x,y)\cdot P_{\left(x,y),(x^{\prime },y^{\prime }\right)}}\right\}, \end{array}$$

in which case (*x'*, *y'*) becomes the new current configuration. Otherwise (*x'*, *y'*) is discarded and (*x*, *y*) remains unchanged. Thus, a sampling algorithm is specified via the neighborhoods N(x,y) and the proposal probabilities *P*_(*x*,*y*),(*x'*, *y'*)_

The acceptance criterion Equation (3) is quite general. By using a symmetric proposal probability matrix, *P*_(*x'*,*y'*) (*x*, *y*) _= *P *_(*x*,*y*), (*x'*,*y'*)_, the relationship simplifies to

(4) $$\alpha ((x,y)\to (x^{\prime },y^{\prime }))=\min\left\{1,\frac{q(x^{\prime },y^{\prime })}{q(x,y)}\right\}.$$

Since the distribution *q *appears only in form of a ratio we need to be able to compute *q*(*x*, *y*) only up to a normalization constant. If *q*(*x'*, *y'*) >*q*(*x*, *y*) we accept the proposal and otherwise the proposal is accepted as new configuration with the finite probability *q*(*x'*, *y'*)/*q*(*x*, *y*). Hence, we need to choose the neighborhood of *x*, *y *such that that the ratio $\frac{q\left(x^{\prime },y^{\prime }\right)}{q\left(x,y\right)}$ is not too far away from one. Otherwise, virtually only proposals that increase the probability are accepted and the sampling procedure gets stuck at local maxima.

Equation (4) and its generalization Equation (3) describe *Markov chains *in the configuration space $Z=\Sigma^{L_{\text{Q}}}\times \Sigma^{L_{\text{S}}}$ with the transition matrix *T*_(*x*, *y*), (*x'*, *y'*) _= *P*_(*x*, *y*), (*x'*, *y'*)_·*α*((*x*, *y*) → (*x'*, *y'*)).

For an appropriate choice of the neighborhoods N(x,y) and of the proposal distribution *P*_(*x*, *y*), (*x'*, *y'*)_, the so-constructed Markov chain is ergodic (each configuration can be reached from any starting configuration with finite probability). Furthermore, one can show that the detailed balance condition

(5) $$q(x,y)T_{\left(x,y)\;(x^{\prime },y^{\prime }\right)}=q(x^{\prime },y^{\prime })T_{\left(x^{\prime },y^{\prime })\;(x,y\right)},$$

which is fulfilled due to the choice of *α*(.) according to Eq. (3), implies that the chain converges towards the desired sampling distribution *q*.

We say that the chain has reached *equilibrium *when convergence has occurred up to numerically negligible error. Thus, if the configuration (*x*, *y*) is sampled after equilibration, it will behave like a sample from *q*. In practice, the exact speed of equilibration is unknown and convergence diagnostics are applied (see below). Several (almost) independent samples are obtained by running the chain further and taking a sample every *k*-th step for sufficiently large *k *to allow time for "forgetting" (i.e., decorreclating from) the state of the last sampled configuration. This time is usually referred as *mixing time*.

### Implementation

In this section we show how the sampling algorithm for pairs of sequences is actually designed, based on the background given in the two preceding sections, such that the tails of the probability distributions for the scores can be addressed. The crucial point of the Metropolis-Hastings update is the choice of an appropriate neighborhood N(x,y) (and the related proposal probabilities) and the computation of the probabilities of newly proposed states *q*(*x'*, *y'*). The neighborhood should be chosen such that the acceptance rate Eq. (3) is between 0.3 and 0.7. We shall factorize the (un-normalized) pmf *q *in two contributions, firstly weights w: ℤ → ℝ^+ ^that assign each score value of interest a weight and secondly the null probability, i.e.

(6) q(x,y)=w(S(x,y))⋅p(x,y).

Note that we will leave *w*(·) undetermined for a moment, until Section "Wang-Landau Sampling". The importance reweighting equation Eq. (2) for *h_s _*is then

(7) $$\begin{array}{c} \text{Prob(}S=s\text{)}=E\;[h_{s}(X,Y)]\\ =\sum_{x,y}h_{s}(x,y)\cdot p(x,y)\\ \approx \frac{1}{Z}\sum_{i=1}^{n}\frac{h_{s}({x^{\prime }}^{\left(i\right)},{y^{\prime }}^{\left(i\right)})}{w(s)} \end{array}$$

with the normalization constant

$$Z=\sum_{s}\sum_{i=1}^{n}\frac{h_{s}({x^{\prime }}^{\left(i\right)},{y^{\prime }}^{\left(i\right)})}{w(s)}$$

For the (HMM) a single distribution of scores is not sufficient: Each query is a member of a certain sub-class characterized by the number of transmembrane regions "# of TM helices" to be determined by the Viterbi algorithm (see below). Thus, each class has its own probability *P_n_*(*s*) = Prob(*S *= *s*, # of TM helices = *n*_TM_). In order to take this property into account, we deal with the joint probability Prob(*S *= *s*, # of TM helices = *n*_TM_). Accordingly, the weights have a two dimensional domain, we write *w*(*s*, *n*). Also *h_s _*in Eq. (7) is replaced by an indicator function *h_s,n _*that depends on two parameters: *h_s,n_*(*x*, *y*) equals 1 if *S*(*x*, *y*) = *s *and # of TM helices of x = *n*_TM_. The sampling distribution is generalized to

(8) $$q(x,y,n_{\text{TM}})=w(S(x,y),n_{\text{TM}})\cdot p(x,y),$$

and the the reweighting relationship reads as

(9) $$\begin{array}{ll} & \text{Prob(}S=s,N=n_{\text{TM}}\text{)}\\ = & E\left[h_{s,n_{\text{TM}}}(X,Y)\right]\\ = & \sum_{x,y}h_{s,n_{\text{TM}}}(x,y)\cdot p(x,y)\\ \approx & \frac{1}{Z}\sum_{i=1}^{n}\frac{h_{s,n_{\text{TM}}}({x^{\prime }}^{\left(i\right)},{y^{\prime }}^{\left(i\right)})}{w(s,n_{\text{TM}})}, \end{array}$$

with, in this case, $Z=\sum_{s,n_{\text{TM}}}\sum_{i=1}^{n}\frac{h_{s}({x^{\prime }}^{\left(i\right)},{y^{\prime }}^{\left(i\right)},n_{\text{TM}})}{w(s,n_{\text{TM}})}$.

Generally the occurrence of two sequences *x *= $x=x_{1}\;...\;x_{L_{\text{Q}}}$ and $y\;=\;y_{1}\;...\;y_{L_{\text{S}}}$ is characterized by the null probability

$$\begin{array}{ll} & p(x,y)=\text{Prob(}X=x,Y=y\text{)}\\ = & f^{\text{query}}(x_{1}\;...\;x_{L_{\text{Q}}})\cdot f^{\text{subject}}(y_{1}\;...\;y_{L_{\text{S}}})\\ = & f^{\text{query}}(x)\cdot f^{\text{subject}}(y). \end{array}$$

This simple factorization allows us to draw proposals for the query and for the subject independently. Hence, for simplicity, a neighboring configuration will leave one of the two sequences unchanged. Thus, for selecting a neighboring configuration, first one of the two sequences is chosen at random with probability 1/2. In the case of (FQPS) the subject is always chosen. Then one sequence is chosen from the neighborhood of the selected sequence, as described next. Formally, this means for (RQGS) and (HMM) we use the factorized proposal densities *P*_(*x*, *y*), (*x'*, *y'*) _= 0.5*P*_*x*, *x*' _**1**_*y*, *y*' _or *P*_(*x*, *y*) (*x'*, *y'*) _= **1**_*x*, *x' *_*P*_*y*, *y' *_(**1**_*y*, *y' *_denotes the indicator function which is only one if *y *= *y'*, 0 else. *P*_*x*, *x' *_denotes the proposal of a single sequence) depending on the choice of sequence in the first step.

### Proposal densities for (FQPS) and (RQGS)

In the simplest case either both sequences are i.i.d. or the query is fixed (to some sequence $\tilde{x}$) and the null-model probabilities of their occurrence factorize, i.e.

(10) $$f^{\text{query}}(x)=\{\begin{array}{ll} f^{\text{iid}}\left(x\right)=\prod_{i=1}^{L_{\text{Q}}}f_{x_{i}} & \text{for (RQGS) and}\\ 1_{\left\{x=\tilde{x}\right\}} & \text{for (FQPS)} \end{array}$$

and of course in both cases

(11) $$f^{\text{subject}}(y)=f^{\text{iid}}(y)=\prod_{i=1}^{L_{s}}f_{y_{i}}$$

**Figure 2:**
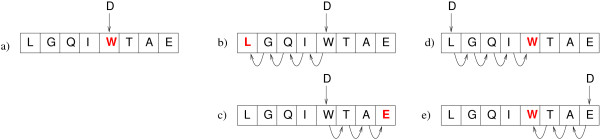
**Monte Carlo moves used in the simulation**. (a) substitution, (b) insertion with left shift, (c) insertion with right shift,(d) deletion with right shift and (e) deletion with left shift.

Due to the factorization that occurs in Eq. (10) it is possible to draw sequences from N(x) such that the detailed balance condition *f*^*iid*^(x) *P*_*x*,*x' *_= *f*^*iid*^(*x'*) *P*_*x'*, *x *_is fulfilled by the following set of Monte Carlo moves (see also Figure 2 and Table 1)

**Table 1:** Monte Carlo operations

operation	resulting sequence
substitution of D at position 5	LGQIDTAE
insertion of D at position 5 with left shift	GQIWDTAE
insertion of D at position 5 with right shift	LGQIDWTA
deletion at position 5 with left shift	LGQITAED
deletion at position 5 with right shift	DLGQITAE

Valid Monte Carlo operations for input sequence *s *= LGQIWTAE (indexing starts with 1). In order to obtain sequences of the same length as *s*, in the case of a deletion a character (D) to be appended at the border has to be specified.

a) substitution of a single symbol at position *k*,

b) insertion of a single new symbol at position *k *with left shift (deletion of the first symbol),

c) insertion of a single new symbol at position *k *with right shift (deletion of the last symbol),

d) deletion of a single symbol at position *k *with right shift and insertion of a single new symbol at the beginning,

e) deletion of a single symbol at position *k *with left shift and insertion of a single new symbol at the end.

Operation a) appears with probability 1/2 and the other ones with probability 1/2 · 1/4 each. This is one possible choice that guarantees detailed balance.

Note that all sequences in N(x) have the same length and each operation involves a replacement of an existing symbol with a newly drawn symbol, in case a) by a direct substitution and in the cases b)-e) indirect via a shift operation. Each position of a sequence has the same probability of being chosen and the replaced symbol is chosen in all cases according to the frequencies *f_σ _*(σ ∈ ∑).

With this construction the Metropolis-Hastings ratio Eq. (3) simplifies to the special case of the Metropolis algorithm, i.e.

$$\begin{array}{ll} & \alpha ((x,y)\to (x^{\prime },y^{\prime }))\\ = & \min\left\{1,\frac{q(x^{\prime },y^{\prime })\cdot P_{\left(x^{\prime },y^{\prime }),(x,y\right)}}{q(x,y)\cdot P_{\left(x,y),(x^{\prime },y^{\prime }\right)}}\right\}\\ = & \min\left\{1,\frac{w(S(x^{\prime },y^{\prime }))p(x^{\prime },y^{\prime })\cdot P_{\left(x^{\prime },y^{\prime }),(x,y\right)}}{w(S(x,y))p(x,y)\cdot P_{\left(x,y),(x^{\prime },y^{\prime }\right)}}\right\}\\ = & \min\left\{1,\frac{w(S(x^{\prime },y^{\prime }))}{w(S(x,y))}\right\}. \end{array}$$

The right part in the second line cancels, because all contributions to *p*(*x*, *y*) and *p*(*x'*, *y'*) where symbols from (*x, x'*) and *y*, *y*) agree cancel directly and for the few remaining letters, the proposal probability in the nominator contains exactly the frequencies of the null probability in the denominator, and vice versa. Thus, the acceptance rate depends only on the score value of the current configuration *S*(*x*, *y*) and the one of the proposal *S*(*x'*, *y'*). Furthermore, it is easy to prove that the detailed balance conditions in Eq. (5) is fulfilled for this chain which in turn implies that the chain converges towards the sampling distribution *q*.

### Proposal densities for the (HMM)

In contrast to the approach presented in the previous section, the generalized method we use here also works for null models that do not allow for direct sampling from N(x) as in the case of i.i.d. sequences. This framework can be summarized by following algorithm:

METROPOLISHASTINGSUPDATE(*x*, *y*, *z*, *p*, *s*, *n*, *w*)

**Input: **Sequences *x*, *y*, a hidden state sequence *z*, the null probability *p*(*x*, *y*) = *f*^query^(*x*). *f*^subject^(*y*), the score *s *= *S*(*x*, *y*), the sub-class *n *and weights *w*

**Output: **Possibly new values for *x*, *y*, *z*, *p*, *s*, *n*.

1: Draw (*x'*, *y'*) ∈ *N*(*x*, *y*)

2: compute *z' *:= *V*(*x*) using V and determine the corresponding class *n'*;

3: compute *p' *:= *f*^query ^(*x'*) · *f*^subject^(*y'*);

4: compute *s' *:= *S*(*x'*, *y'*) using A.

5: Compute $\alpha :=\frac{w[s^{\prime },n^{\prime }]\cdot p^{\prime }\cdot P_{x^{\prime },x}}{w[s,n]\cdot p\cdot P_{x,x^{\prime }}}$.

▷ *Designed such that*

*p*(*x'*, *y'*) · *P*_(*x'*, *y'*), (*x,y*) _= *p*(*x*, *y*) · *P*_(*x*,*y*),(*x'*, *y'*)_

6: With probability min {1, *α*}:

Let (*x*, *y*, *z*, *p*, *s*, *n*)← (*x'*, *y'*, *z'*. *p'*, *s'*, *n'*)

7: **return **(*x*, *y*, *z*, *p*, *s*, *n*)

The algorithm is applicable to all models that allow for a rapid calculation of the null probabilities *f*(·). Sequence models based on HMMs fall into this class. In the following we brie y describe this framework. A detailed discussion can be found in the specialized literature on the topic [2,4].

In the probabilistic framework of HMMs one assumes a sequence of "observed" symbols (the protein sequences here) which is generated conditioned on a sequence of "hidden" states. For the case of TM proteins, the state corresponds to the physical region where the corresponding amino acid is located in, as detailed below. Within a modeling using HMMs, this state sequence, also called *path*, follows a simple Markov chain. The actually generated symbols are connected to the hidden states by conditional "emission" probabilities. More formally, a HMM consists of

• a finite set ∑ of (output) symbols (in our case the amino acid alphabet),

• a finite set Γ of (hidden) states,

• initial state probabilities *π*_*μ *_for all *μ *∈ Γ with ∑ _*μ*∈Γ _*π*_*μ *_= 1,

• emission probabilities $p_{\sigma }^{\mu }$ in each state *μ *∈ Γ Σ and for all *σ *∈ ∑ with $\sum_{\sigma \in \sum }p_{\sigma }^{\mu }$ = 1 for all *σ *∈ ∑,

• a stochastic transition probability matrix *P *= (*p*_*μ*,*τ*_)_*μ*,*τ *∈ Γ_, i.e. $\sum_{\tau \in \Gamma }p_{\mu ,\tau }$ = 1 for all *μ *∈ Γ.

Given these model parameters, the "most natural" application of a HMM is to generate a sequence of hidden states by a stochastic process and, in parallel, to generate a random sequence of symbols given the generated states. Hence, the stochastic process describes pairs of states and symbols. But also given a fixed state *Z *= *Z*_1 _... *Z_L_*, the symbol sequence *x *= *x*_1 _... *x_L _*is a stochastic process, furthermore the opposite case of a fixed sequence of output symbols, the state sequence is a stochastic process.

For the Monte Carlo sampling as needed here, it is not possible to simulate a HMM directly to generate symbol sequences, since importance sampling changes the underlying sequence probabilities. Nevertheless, one still needs to compute the probabilities *f*^HMM^(*x*) for the Monte Carlo acceptance procedure, i.e. the probabilities that *x *is the observed symbol sequence generated by the HMM using any feasible state sequence. These probabilities can be computed in O(*L *· |Γ|^2^) time using the well known *forward algorithm *as described in the following. One introduces the auxiliary variables *f_μ_*(*i*), which correspond to the probability that the subsequence *x*_1 _... *x_i _*is generated by the HMM given that the last state variable *Z_i _*has the value *μ*, i.e. *f_μ _*(*i*) = Prob(*X*_1 _... *X_i _*= *x*_1 _... *x_i_*|*Z_i _*= *μ*). The overall probability is then *f*^HMM^(*x*) = ∑ _*μ*∈Γ _*f_μ _*(*L*). The probabilities *f_μ _*(*i*) can be determined by the recursion

(12) $$f_{\mu }(i)=p_{x_{i}}^{\mu }\sum_{\tau \in \Gamma }f_{\tau }(i-1)p_{\tau ,\mu }$$

with initial conditions $f_{\mu }(1)=\pi_{\mu }p_{x_{1}}^{\mu }$.

Within the same time complexity the *Viterbi algorithm *V computes the most probable state path for a given sequence of observations, that is

$$\begin{array}{ll} & z_{1}\ldots z_{L}=V(x_{1}\ldots x_{L})\\ = & \underset{{\bar{z}}_{1}\ldots {\bar{z}}_{L}\in \Gamma^{L}}{\text{argmax}}\text{Prob}\left(Z_{\text{1}}\ldots Z_{L}={\bar{z}}_{\text{1}}\ldots {\bar{z}}_{L}\text{|}x_{\text{1}}\ldots x_{L}\right). \end{array}$$

For this purpose one uses a different set of auxiliary variables: Let *v_μ _*(*i*) be the probability of the most probable path ending in state *μ *∈ Γ with observed partial output sequence *x*_1_, ..., *x_i_*. These values can be computed recursively by

(13) $$v_{\mu }(i)=p_{x_{i}}^{\mu }\underset{\tau \in \Gamma }{\max}\{v_{\tau }(i-1)p_{\tau ,\mu }\}$$

with boundary condition $v_{\mu }(1)=\pi (\mu )\cdot p_{x_{1}}^{\mu }$. Note that these probabilities are not normalized, in particular $\sum_{\mu \in \Gamma }v_{\mu }(i)\leq 1$. The missing normalization is no problem, since we are interested only in the most probable path, which is reconstructed by back-tracking [2].

For the approach discussed in this section, the subject sequences are drawn almost as above, see below. The HMM approach we use to sample transmembrane queries is the TMHMM developed by Sonnhammer et. al. [25]. In this setting, the states are (structural) domains. Some of them are "tied", which means that they share the same emission probabilities. They are classified into seven groups:

• Helix core,

• two different groups of caps on either side,

• loops on the cytoplasmic side,

• short and long loops on the non-cytoplasmic side,

• globular domains.

**Figure 3:**
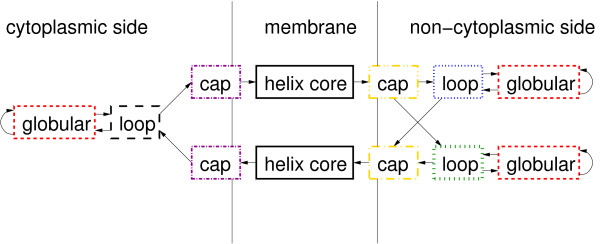
**The layout of the HMM for transmembrane proteins**. The layout of the HMM for transmembrane proteins according to Sonnhammer et.al. [25]. Each box corresponds to a group of states. For example the helix-core block consists of 25 internal states. Line type of boxes represent different emission probabilities. For more details we refer the reader to the original publication.

The internal structure of the helix core and loop module allows modeling different lengths of the corresponding protein domain by assigning jump probabilities. The globular domains have a self-looping structure and hence may also have various lengths. The other modules have fixed lengths. The overall number of model parameters is 216. Figure 3 shows the actual layout of TMHMM. Each box represents a group of "tied" states. The states corresponding to "helix core" represent the transmembrane helices that connect states of the cytoplasmic side and the non-cytoplasmic side of the membrane. The prediction of the positions of the "helix core" states determines the loci of the special purpose scoring matrix SLIM for position specific alignment (see Figure 1).

The following Metropolis-Hastings update consists of two steps: First, the proposal of a new configuration from the neighborhood N(x) is made by inserting/replacing symbols with equal weights $f_{\sigma }=\frac{1}{\left|\sum \right|}$ for all σ ∈ ∑ using one of the five Monte-Carlo moves described above. The acceptance ratio Eq. (3) in that case is given by

(14) $$\begin{array}{ll} & \alpha ((x,y)\to (x^{\prime },y^{\prime }))\\ = & \min\left\{\begin{array}{l} 1\\ \frac{w(S(x^{\prime },y^{\prime }),{n^{\prime }}_{\text{TM}})f^{\text{query}}(x^{\prime })f^{\text{subject}}(y^{\prime })}{w(S(x,y)n_{\text{TM}})f^{\text{query}}(x)f^{\text{subject}}(y)} \end{array}\right\} \end{array}.$$

The current and new number of TM regions *n*_TM _and *n'*_TM _are determined by the Viterbi algorithm applied on the sequence *x *and *x' *respectively. The calculation of the query probabilities is based on the TMHMM. The subject sequence probability is simply calculated according to Eq. (11). Note that, in each step, one of the two probabilities cancels, because only one of the two sequences is changed within each step, as above.

This approach allows us to sample noni.i.d. sequences with appropriate weights and to predict transmembrane helical regions that can be used in the position specific alignment scheme (as described in [23]) even for random sequences.

### Wang-Landau sampling

The idea of importance sampling is to choose the weights *w*(·), such that the drawn events in the region of interest have a high probability to occur in the simulation. Ideally, *P*(*S*) is already known and in that case one might choose *w*(*S*) ∝ 1/*P *(*S*) on the entire range of interest. Then all states are visited with equal probability, and hence a at score histogram is achieved in the limit of infinite sample size. Still, for practical applications with finite sample size, the distribution of scores can be sampled with high accuracy over a large range of its support. This idea refers back to statistical physics and it is known as "generalized ensemble" or " flat histogram" methods. In the following we will denote this weights by *w*^flat^.

Of course the *true P*(*S*) is unknown and the method requires some guesses which approximate *w*^flat ^to a suitable accuracy. The achieved score histogram becomes only approximatively flat. The true (unknown) distribution can then be estimated by reweighting the histogram of visited states using the importance sampling formula Eq. (7) for *h_s_*.

Many iterative sampling schemes to achieve initial guesses had been developed in the 1990ies, for example entropic sampling [30], multicanonical sampling [31] and later transition matrix Monte Carlo [32-34], only to mention a few. Here we use the Wang-Landau algorithm [35,36] to approximate *w*^flat ^as input for Metropolis-Hastings sampling.

The Wang-Landau algorithm explicitly violates detailed balance by dynamically updated weights depending on the visited states in the following way: First, a score range of interest [*S*_min_, *S*_max_] is chosen. The algorithm basically employs a histogram *H*(*S*) and weights *w*(*S*) defined on the desired score range. For more complicated models such as the (TMHMM), these objects are two-dimensional depending on the score *S *and the class *n*_TM_, i.e. *H*(*S*, *n*_TM_) and *w*(*S*, *n*_TM_). Furthermore, real valued parameters *ϕ_i _*> 1 are used in each iteration *i*. Initially, the histogram values *H*(*S*, *n*_TM_) are set to 0 in the desired range and all weights *w*(*S*, *n*_TM_) to a constant, say 1. For the first iteration, *i *= 0, *ϕ_i _*can be as large as *e*^1^. Then, a simulation is performed using acceptance ratio Eq. (12) or Eq. (14). After each step, corresponding to one step of a (biased) *random walk *in the configuration space, *w*(*S*, *n*_TM_) is updated as *w*(*S*, *n*_TM_) ← *w*(*S*, *n*_TM_) × *ϕ_i_*, where *S *is the current score value and *n*_TM _the sub-class of the current state. Also the histogram H is updated by one *H*(*S*, *n*_TM_) ← *H*(*S*, *n*_TM_) + 1. In the literature this is often continued until an "approximately flat histogram" is achieved. A possible flatness criterion might be $H(S,n_{\text{TM}})>0.6\cdot \frac{1}{S_{\max}-S_{\min}+1}\sum_{S^{\prime }=S_{\min}}^{S_{\max}}H(S^{\prime },n_{\text{TM}})$ for all *S*, *n*_TM_. Once the histogram is " flat", *ϕ *is decreased by the rule $\phi_{i+1}\leftarrow \sqrt{\phi_{i}}$ and all entries of the histogram *H *are set to 0 again, while *w *is kept for the next iteration. Note that the application of a flatness criterion is not essential for the good performance of the algorithm. It is enough to guarantee that all values of *S *have been visited, for example by requiring that the random walker has cycled several times through the interval of interest [*S*_min_, *S*_max_].

To summarize, we have the following recipe:

WANGLANDAU(*w*, *ϕ*, *ϕ*_final_, *N*)

**Input: **Initial guess *w*[*s*, *n*], initial and final modification factors *ϕ*, *ϕ*_final_, number of samples for production run *N*

**Output: **Histogram of visited scores, *H*(*s*, *n*):= number of samples with score *s *and class *n *and weights used in the production run *w*(*s*, *n*) for all *s *and *n*

1: ▷ *Initialize and estimate w*[*s*, *n*]

2: Pick any *x*, *y *∈ X and compute its null probability *p *:= *f*^query^(*x*) · *f*^subject^(*y*);

3: compute *s *:= *S*(*x*, *y*) using A

4: compute *z *:= *V *(*x*) using V and determine corresponding class *n;*

5: **while ***ϕ *>*ϕ*_final _**do**

6:   *H*[*s'*, *n'*] ← 0 for all possible score values *s' *and classes *n'*

7:   **while ***H*[*s'*, *n'*] is not at **do**

8:      (*x*, *y*, *z*, *p*, *s*, *n*) ←

      METROPOLISHASTINGSUPDATE (*x*, *y*, *z*, *p*, *s*, *n*,*w*);

9:      *H*[*s*, *n*] ← *H*[*s*, *n*] + 1; *w*[*s*, *n*] *w*[*s*, *n*]/*ϕ*;

10:   **end while**

11:   $\phi \leftarrow \sqrt{\phi }$

12: **end while**

13: ▷ *Obtain N samples from q and their score counts/histogram*

14: *H*[*s'*, *n'*] ←0 for all possible score values *s' *and classes *n'*

15: **for ***i *= 1..*N ***do**

16:   *H*[*s*, *n*] ← *H*[*s*, *n*] + 1

17:   **repeat**

18:      (*x*, *y*, *z*, *p*, *s*, *n*) ←

      METROPOLISHASTINGSUPDATE (*x*, *y*, *z*, *p*, *s*, *n*,*w*);

19:   **until **mixing has occurred

20: **end for**

21: **return **counts *H*, weights *w*.

Due to the decreasing rule $\phi_{i+1}\leftarrow \sqrt{\phi_{i}}$, the modification factor *ϕ *converges towards 1. The simulation is stopped when *ϕ *reaches a chosen threshold value which is close to 1. It turned out that in our case the range from *ϕ*_0 _= exp(0.1) ≈ 1.105 to *ϕ*_final _= exp(0.0002) ≈ 1.0002 has been proven valuable.

Since detailed balance is violated explicitly, the convergence of the algorithm can not be proven. For this reason one should always use the Wang-Landau part as a precomputation step just to obtain weights suitable *w*(*S*). After this, one performs a simulation with *ϕ *= 1 for data production, which corresponds to the Metropolis-Hastings algorithm.

#### Improvements

**Figure 4:**
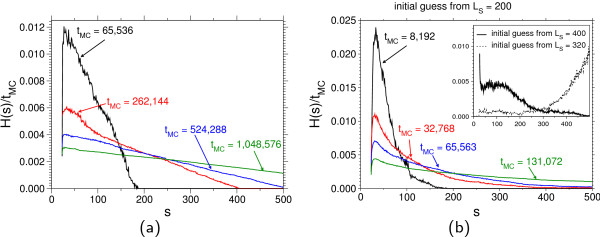
**Dynamics of the Wang-Landau algorithm**. Typical time evolution of the histogram of visited states when starting with different initial guesses. The model parameters are *RQGS *with *L_Q _*= *L_S _*= 348. The weights have been updated dynamically with modification factor *ϕ *= exp(0.1) ≈ 1.105. (a) *w*(*s*) = 1 for all *s*. The Markov chain converges relatively slowly. (b) *w*(*s*) ≈ 1/Prob(*S *= *s*|*L*_*Q *_= 348, *L*_*S *_= 200) has been used as an initial guess. The histogram becomes flatter within remarkable less computational effort. Inset: a detailed balance simulation (*ϕ *= 1 during the simulation of 1, 048, 576 steps) with initial weights that are close to the inverse target distribution. Though the histograms are not "flat", each score value on the interval [23, 500] has been visited. The estimate from this data can be used in a longer production run.

Of course there is much room for improvement. For example, consider the time evolution of the histogram *H*(*S*) for (RQGS) with *L_Q _*= *L_S _*= 348 up to *S*_max _= 500 with Prob(*S *= *S*_max_) ≈ 10^-65 ^in Figure 4a.

When starting with an initial guess *w*(*S*) = 1 for all *S *∈ [23, 500], the random walker needed about 5.8 × 10^5 ^Monte-Carlo steps for a *round trip*, i.e. to move from the lowest score *S*_min _= 23 to the highest one *S*_max _= 600 and back. The duration of a round trip is a measure of the mixing time of the corresponding Markov chain. Hence, the shorter the round trip is time, the faster the chain convergences. During the first round trip, the weights have been improved such that the second round trip (and further round trips) needed only 13% of the computational effort of the first one. Once the random walker has performed its first round trip, the typical round trip time does not change significantly. This tight bottleneck in the very early stage of the algorithm can be overcome by suitable initial guesses of *w*. In Figure 4b the time evolution of the same parameter set (RQGS with *L_Q _*= *L_S _*= 348) is shown except for the choice of the weights, which have been chosen as *w*(*s*) ≈ 1 = Prob(*S *= *s*|*L_Q _*= 348; *L_S _*= 200), i.e. from a previous simulation of a different but similar setup. One observes that the histogram becomes "at" within a much smaller amount of Monte-Carlo steps. Furthermore, the first round-trip time decreases to 1.3 × 10^5 ^(i.e. 22% of the value for the naive guess *w*(*s*) = 1). From a practical point of view, this allows us to save computing time for two distributions with close by parameters (e.g. close by sequence lengths). One can use the results of one distribution as input for the second one. With this approach we may also explore the parameter space successively. In some cases it is sufficient to run a short batch run with the weights of a close by distribution and *ϕ *= 1, i.e. a detailed balance simulation, and then apply importance reweighting and use the so obtained approximation of *P*(*s*) for a longer production run. This kind of procedure is shown in the inset of Figure 4b: The detailed balance simulations were performed with *L_Q _*= *L_S _*= 348, whereas the weights *w*(*s*) came from a simulation with *L*_S _= 320 and *L*_S _= 400, respectively. The result shows that the histograms are not " flat" at all, but the distributions were close enough to visit all score values on the range of interest. In this successive way of iterations a broad range of the parameter space is accessible.

#### Estimation of the statistical error

Statistical analysis of Markov-chain Monte-Carlo data requires a careful inspection of correlation effects because the events depend on the history of the chain. This correlations vanish within a typical timescale: Events that are separated by a sufficient number of steps can be assumed to be independent. However, since Monte-Carlo methods are only approximative, an assignment of statistical errors are requisite. In this study we used Flyvbjerg and Peterson's [37] blocking method to estimate the error.

## Results

To our knowledge we present the first highly accurate score statistics for alignments with position-specific scoring schemes. The alignment scores were calculated with the standard Smith-Waterman algorithm with the BLOSUM62 matrix for the (RQGS) and a bipartite version BLOSUM62/SLIM for (FQPS) and (HMM) (see Figure 1). For the a fine gap costs we have chosen the standard values with a gap-open penalty of 12 and a gap-extension penalty of 1, and UniProt symbol frequencies for i.i.d. sequences.

**Figure 5:**
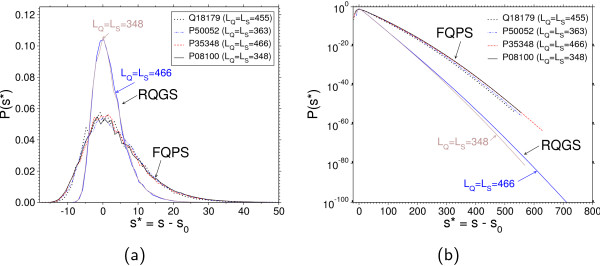
**Score distributions for (RQGS) and (FQPS) models**. Score distributions for (RQGS) (classical) and (FQPS) models where the subject length equals the query length. In order to compare the shape, the distributions have been shifted by the center *s*_0_. (a): Linear view; all distributions from the (RQGS) agree outside the tails (only two lengths are shown). The shape of the (FQPS) distributions is more variable. (b): Logarithmic view; significant differences between the two models appear in the tail of the distribution. High scores are more probable for the (FQPS) alignment. Furthermore the curvature, i.e. the deviation from the Gumbel form, is much larger for (FQPS) than for the classical model.

We discuss four different transmembrane proteins as queries (see Table 2) in the (FQGS) scheme. The results are shown in Figure 5, where the distributions of (FQGS) and (RQGS) are compared against each other. The subject lengths are set to the query lengths. For the production run of one distribution in Figure 5 (*L*_Q _= *LS *= 348) 16,777,216 Metropolis-Hastings updates have been performed. This took about 16 hours on an Intel Pentium 4 with 3.4 GHz. The performance of the corresponding HMM is weaker for three reasons: Firstly, we are interested in a joint distribution for that we need more samples. Secondly, more proposals are rejected from the sampler due to the HMM-weights and finally the computation of the forward-probabilities requires additional floating point operations. The computation of 16,777,216 Metropolis-Hastings updates for this model costs about 45 CPU hours. We use an 8 times larger sample size in order to account for the first drawback. Hence, we put an overall computational effort on this model, which is 23 times as large as for (FQGS) and (RQGS) (apart from the Wang-Landau iterations).

**Table 2:** A selection of transmembrane proteins

ID	AC	Description	Organism	Length
OPSD_HUMAN	P08100	Rhodopsinm	H. sapiens	348
AGTR2_HUMAN	P50052	type-2 angiotension II receptor	H. sapiens	363
YXX5_CAEEL	Q18179	putative neuropeptide Y receptor	C. elegans	455
ADA1A_HUMAN	P35348	Alpha-1A adrenergic receptor	H. sapiens	466

A selection of transmembrane proteins. ID: UniProt identifier; AC: accession number.

Here we observe in Figure 5b that on the log scale the curvature of the tail of the distribution, i.e., the deviation from the exponential tail of the pure Gumbel form Eq. (2), is more pronounced in the (FQPS) model: Significant differences of shapes already show up in the high probability region, which is accessible by simple sampling (Figure 5a). The (RQGS) distributions for different lengths match almost perfectly (only two lengths are shown), whereas the shape of the (FQPS) distributions varies slightly with the sequence type. This supports the observation of Müller et.al. [23] that position-specific scoring in connection with a fixed query sequence may better discriminate between different sequences than the standard approach where two random sequences are compared with position-independent scoring matrices.

The asymptotic theory for i.i.d. sequences predicts an EVD of the form of Eq. (2). The parameters λ > 0 and *c *> 0 depend on the score matrix, on the symbol frequencies *f*, and on the query and subject sequence lengths *L*_Q _and *L*_S_. Altschul and Gish [16] pointed out that asymptotic results where *c *= *KL*_Q_*L*_S _(with *K *> 0 a constant) need to be corrected by using effective sequence lengths. An alignment may extend to the end of either sequence and the score will be distorted towards lower values and high scores become less probable. In the limit of infinite sequences this effect vanishes and the tail of the Gumbel distribution can be understood as an upper bound for finite sequences. Indeed, we clearly see that the curves in Figure 5b are not straight lines in the right tail, but have negative curvature.

A better t to the empirical distribution is obtained by determining parameters *s*_0_, λ > 0, λ_2 _> 0 for a "modified" Gumbel distribution with

(15) $$\log\text{Prob}(S=s)=\log(\lambda )-\lambda (s-s_{0})-\lambda_{2}{\left(s-s_{0}\right)}^{2},$$

where *s*_0 _can be interpreted as the center of the distribution. This corresponds to a EVD multiplied with a Gaussian correction factor, given by the last term. The parameter λ_2 _is generally small (and thus shows its effect only in the far right tail). It vanishes for sequences of equal length as the length tends to infinity. Previously, such a correction has been proposed for (RQGS) statistics and has been computed for different parameter sets of BLOSUM62 and PAM250 with a ne gap costs [17,18].

More pronounced differences are seen in the behavior of the tail (Figure 5b), which is only accessible via importance sampling approach. The difference between the probabilities spans several orders of magnitude; hence a wrong choice of the model would falsify the estimation of significance drastically. Most importantly, the pmf obtained using the position-specific scoring is considerably curved. Thus, using EVDs from fits to data of the high-probability region is even more questionable here than in the (RQGS) model, where the pmf is almost a straight line. Note that for the (RQGS) model, previous simulations [18] have already shown that for the special case of *L*_S _= *L*_Q_, the pmf converges for large sequence length indeed to an EVD.

Note that the Gaussian correction for local alignment parameterized by λ_2 _is purely heuristic. Looking at the data, the shape in Figure 5b looks similar to the one of the Tracy-Widom distribution [38]. Interestingly, Majumdar and Nechaev [39] as well as Priezzhev and Schütz [40] obtained analytically the Tracy-Widom distribution as the asymptotic distribution for the model of the longest common subsequences which is closely related to global alignment. Also, Sardiu, Alves and Yu [41] observed that the the statistics of the score fluctuations of global alignment in the large probability region is compatible with the Tracy-Widom distribution. There could be a connection to our results, because in the rare-event tail, alignment lengths are of the order of the sequence lengths, hence the alignment is effectively global. Nevertheless all our results are obtained for finite sequence lengths. In contrast, distributions like Gumbel or Tracy-Widom are obtained in the asymptotic limit of infinite sizes of the underlying systems. Since finite-size corrections are hard to obtain, or even unknown, we do not attempt to determine the "true" shape of the distribution and are satisfied by our heuristic formula.

**Figure 6:**
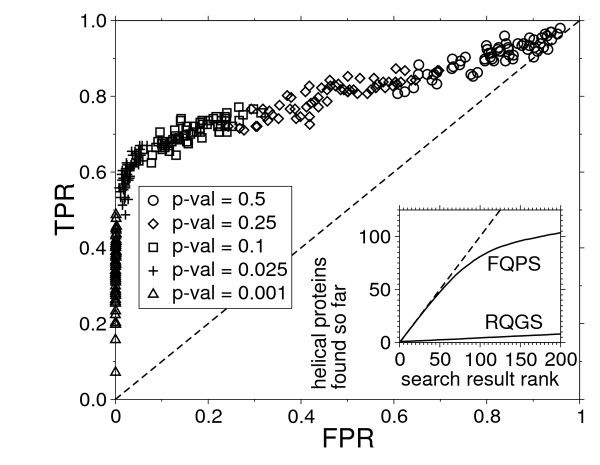
**Retrieval performance for the (FQPS) statstics**. ROC curves (true positive rate vs. false positive rate) when searching TM proteins from the ASTRAL reference set against the complete ASTRAL set. Different symbols indicate different p-value thresholds being used. Inset: sensitivity for (FQPS) compared with BLAST search. The plot shows the averaged number of observed helical proteins as a function of the rank in the result set.

Next, we discuss the usefulness of the (FQPS) statistics in terms of retrieval performance. For this purpose we considered the ASTRAL compendium [42] version 1.75 with less than 40% identity to each other. It contains a set of reference proteins classified hierarchically based on their tertiary structure. It is a subset of the SCOP database with removed redundancy. The main hierarchy levels in the SCOP classification scheme are *class, fold*, *superfamily, family*. Proteins in the same class share the same type of secondary structure, whereas the fold level describes more specific the arrangement of the secondary structure. As the position specific scoring scheme is designed to be more sensitive to discriminate transmembrane proteins against others, its performance can be measured by searching a collection of transmembrane proteins from the ASTRAL set against the complete set. From the 10569 sequences in the database we have chosen 63 sequences which are classified *as Membrane and cell surface proteins and peptides*. For this collection we predicted the membrane regions using TMHMM. Each of those queries were searched against the complete set and ranked according to the p-value on the basis of the (FQPS) statistics. The p-value threshold under which we regard a hit as significant controls the so called receiver operating characteristic (ROC), i.e. the relationship between sensitivity vs. specificity. A transmembrane protein that appears below an p-value threshold is referred as a *true positive *observation. Accordingly, proteins of all other SCOP classes below that value are *false positives*. The ROC space can be explored by changing the p-value threshold. A small threshold produces less false positives but we may miss some hits. A larger value leads to more false positives. The ROC is usually illustrated by plotting the true positive rate (TPR = true positives/positives), or the sensitivity, against the false positive rate (FPR = false positives/negatives), 1- specificity. The result for the search characteristics for (FQPS) is shown in Figure 6. We did the same experiment for a BLAST search. In this case all observations are located in left bottom corner in the ROC space. This can be explained by the fact that we have only considered the highest ranked results below the E-value threshold of 10 and many positives were beyond that value. This can also be seen in the inset of Figure 6 where we show the average number of membrane proteins found so far as a function of the rank in the result set. The line of slope 1 for (FQPS) at the begin of the result list means that virtually all hits are found in the correct SCOP class. In contrast, BLAST ranks transmembrane proteins at a high positions only randomly. Hence, the (FQPS) clearly outperforms the (RQGS) statistics.

The ROC curve in Figure 6 show the usefulness of the (FQPS) statistics for retrieval performance, but the extreme small p-values where the the modification factor λ_2 _plays a role are not essential for this purpose. The modified Gumbel statistics however affect a possible ranking of database search results, especially for sequences of different lengths. To illustrate this, we used BLAST to retrieve homologs of our four example proteins from the current Swissprot database. The scores were recomputed via the position specific Smith-Waterman algorithm for (FQPS). We computed the corresponding p-values from our simulation data and ranked the result set by the p-value based on

1. the Gumbel distribution (λ_2 _= 0) and

2. the accurate distribution (λ_2 _> 0).

For subject sequence lengths that are not directly governed by our simulation directly we used interpolated fit parameters. In Table 3 we illustrate that the there are subjects whose relative order in the result set is switched when using the more accurate (FQPS) score distribution in contrast to the BLAST E-value. This result might be important for applications of protein classification where the specific ranking of high scoring proteins is particularly important. However, on a global level the order of the hits persists, signaled by Kendall's rank correlation [43]. When comparing the order obtained between the cases λ_2 _= 0 and λ_2 _≠ 0 we measured a rank correlation $\hat{\tau }$ = 0.986 for the query P08100. A τ of exactly 1 means identical, 0 unrelated, and, -1 exactly the reverse ranking. The rank correlation between the original BLAST ranking and the one based on the accurate p-values is $\hat{\tau }$ = 0.737.

**Table 3:** Change of ranking when using the modified Gumbel distribution

	FQPS λ_2 _= 0				FQPS λ_2 _≠ 0			
Query	rank	Subject	L_S_	p-value	rank	Subject	L_S_	p-value
P08100	433	Q90456	287	1.1 × 10 ^-21^	445	Q8N6U8	529	7.2 × 10 ^-29^
*L*_Q _= 348	476	Q8N6U8	529	2.1 × 10 ^-21^	483	Q90456	287	2.1 × 10 ^-28^

P50052	79	P32250	308	1.1 × 10 ^-37^	64	P34975	380	1.2 × 10 ^-57^
*L*_Q _= 363	100	P34975	380	1.8 × 10 ^-37^	111	P32250	308	1.3 × 10 ^-56^

Q18179	772	P18901	446	2.2 × 10 ^-21^	790	P79291	228	9.2 × 10 ^-27^
*L*_Q _= 455	837	P79291	228	1.1 × 10 ^-20^	794	P18901	446	9.8 × 10 ^-27^

P35348	825	Q8HYN8	297	9.8 × 10 ^-24^	826	O70432	167	5.2 × 10 ^-30^
*L*_Q _= 466	937	O70432	167	1.3 × 10 ^-21^	847	Q8HYN8	297	1.9 × 10 ^-29^

Examples of blast hits for the four proteins used for FQPS. The original result sets have been re-ranked according the the FQPS statistics. Left column: Gumbel assumption (λ_2 _= 0). Right column: Modified Gumbel distribution (λ_2 _≠ 0).

**Figure 7:**
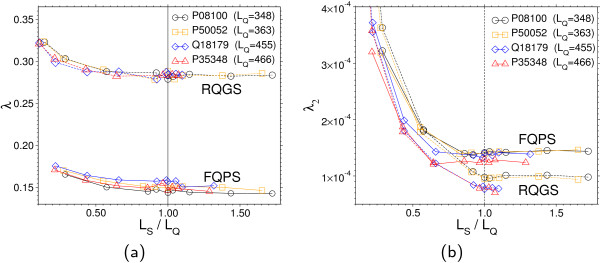
**Fit parameters for (RQGS) and (FQPS) models**. Dependence of the modified Gumbel parameters on the subject/query length ratio *L*_S_/*L*_Q_. The vertical line corresponds to Figure 5, where *L*_S _= *L*_Q_. (a): λ describes the bulk of the distribution (see Figure 5a) left). For *L*_S _>*L*_Q_, λ varies only slightly in the subject length. (b): The parameter λ_2 _characterizes the curvature of the pmf in the tail (see Figure 5b). Large differences between (RQGS) and (FQPS) show up in the case where *L*_S _>*L*_Q_. λ_2 _becomes subject-length independent for *L*_S _>*L*_Q_.

To investigate the impact of dissimilar query and subject lengths *L*_Q _and *L*_S _on the parameters of the modified Gumbel distribution, we vary *L*_S _and consider the parameters λ and λ_2 _as functions of the ratio *L*_S_/*L*_Q _(see Figure 7). The large gap between the values of λ for the two different models reflects the qualitative difference of the shape in the high probability regime. We see that in the (RQGS) model, λ is virtually independent of the query and the sequence length. However, in the model (FQPS), λ varies with each individual query, as expected. For λ_2 _one has to distinguish between *L*_S _<*L*_Q _and *L*_S _>*L*_Q_. In the first case, λ_2 _decreases, which is not surprising, since the correction term describes a finite-size effect and should vanish for increasing sequence lengths.

Once the subject length exceeds the query length, the search space is still growing, but the finite length of the query enforces subject size independent edge effects.

**Figure 8:**
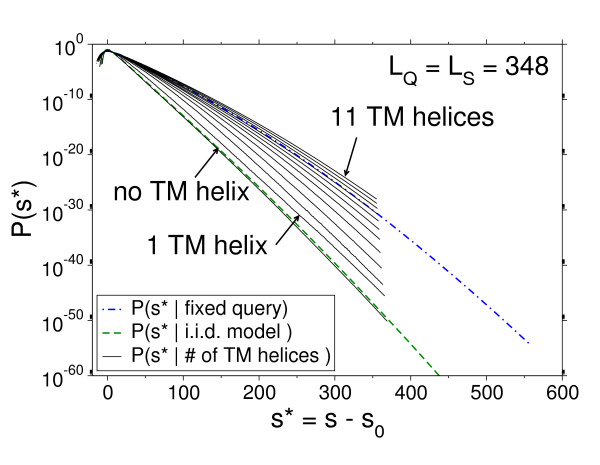
**Score distributions for different alignment models**. Score distributions for different alignment models (i.i.d., fixed query and TMHMM) with *L*_S _= *L*_Q _= 348. The distributions for the (HMM) have been obtained from the joint distribution.

For the (HMM), we approximate the score distribution within each class (number of helices = *n*). The shape of the distributions clearly agrees with the curvature for (RQGS) and (FQPS), and the modified Gumbel distribution could be fitted (see Figure 8) when the number of helices was not too small. This is indicated by a large reduced χ^2 ^value for distributions with a small number of helices. Also a visual inspection of the fit to the data supports this argument.

**Figure 9:**
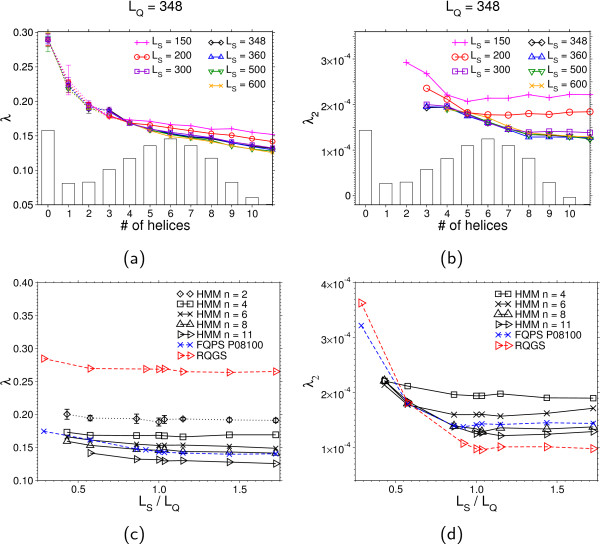
**Fit parameters for different alignment models**. Fit parameters for score distributions *P *(*S*|# of helices) for the (HMM) with a fixed query length *L*_*Q *_= 348 and various subject lengths *L*_*S*_. Both shape parameters λ and λ_2 _decrease with increasing number of helices. The dependency on the subject length is stronger for λ_2 _than for λ. For *L*_*S *_>*L*_*Q *_the dependency of λ_2 _on the subject length is only of marginal order. The bars show the distribution of the number of transmembrane helices obtained by direct simulations of the (HMM). (c),(d): The *L*_*S*_/*L*_*Q *_dependency of λ and λ_2 _extracted from the same data as (a),(b). The lines are guide to the eyes only. Dashed lines show the corresponding scaling behavior for the (FQRS) and (RQGS) models. The result for *n *= 2, that has been obtained from the high probability regions (see text), is indicated by dotted lines.

The rare-event tail shows clear differences between the different sub-classes of the model over several orders of magnitude. In Figure 9 the dependency of the fit parameters on the respective subclass of the model (Figure 9a and Figure 9b) as well as the dependency on the ratio *L*_S_/*L*_Q _(Figure 9c and Figure 9d) is shown. Note that for distributions that are not well described via Eq. (15), we only fitted the data in the high probability region. Those data points are left out in the plot for λ_2 _in Figure 9b and are connected by dotted lines in Figure 9a.

In analogy to (RQGS) and (FQPS), the curvature remains constant when *L*_S _>*L*_Q_. Regarding the dependence on the number of helices, the curvature decays with increasing number of transmembrane regions and then approaches an approximate constant value. Numerical values are provided in the Appendix for reference.

## Discussion and conclusions

We have presented a simple universal numerical method to accurately sample the far right tail of the score distribution of various sequence comparison algorithms. It appears to be the first method that is applicable to all classical local alignment statistics, query-specific and position-dependent score statistics, HMM calibration, statistics of normalized alignments, and many more. To sample the distribution using computer simulations, we use Markov-chain Monte Carlo simulations, in particular the Wang-Landau approach is connection with the Metropolis-Haistings algorithm. Apriori, the Wang-Landau approach does not require any assumption on the shape of the distribution (for example the parameters of the Gumbel distribution). The parameters can be estimated a posteriori by fitting the simulated distribution to an appropriate parametric form like Eq. (15). Here, we observed that for the (FQPS) model, the Gumbel distribution should be replaced by a more negatively curved one.

The method has a disadvantage: Because of the high number of samples required for non-parametric estimation of the distribution, it can presently not be used in on-line database search web services, such as a BLAST server. For example, generating the 16,777,216 samples for Figure 5 (*L*_Q _= *L*_S _= 348) took approximately 16 hours on an Intel Pentium 4 with 3.4GHz.

This is not as bad as it seems, though: Both the implementation and the design of the Markov chain have much room for improvement, e.g. we can choose different neighborhoods *N*(*x*) and optimize the weights in the generalized ensemble [44,45].

While this still prohibits interactive use, we see a lot of potential for our method to provide an improved version of the hmmcalibrate tool [22] and to explore the statistics of normalized sequence alignment [7].

During the preparation of this manuscript we came aware of a new related importance sampling method which is suitable for efficient p-value computations for alignment statistics [46]. It makes use of simultaneous backward sampling of alignments and sequences. So far this method was applied to i.i.d. sequences but it should be possible to extend it to more complex model as well. We have tested it for the (FQPS) model as well. For the joint distribution of score and number of helices one would have to sample simultaneous the alignments, sequences and the hidden state sequence of the TMHMM.

## Authors' contributions

SW developed the simulation program for (FQPS) and HMM based on an earlier version [18], ran the simulations and performed data analysis. SR and AKH designed the project. IH and SW developed the details for the TMHMM. All authors contributed to the manuscript and approved the final manuscript.

## Appendix: modified Gumbel parameters

Table 4 and Table 5 show numerical values for the parameters λ, λ_2 _and *K *of the modified Gumbel distribution Eq. (15). These are visualized in Figure 7 and 9 in the body of the paper.

**Table 4:** Fit parameters for (FQPS) and (RQGS)

		FQPS			corresponding RQGS		
***L***_**Q**_	***L***_**S**_	λ	10^4 ^λ_2_	*K*	λ	10^4 ^λ_2_	*K*
P08100	50				0.3016 ± 0.40%	7.5741 ± 0.77%	0.0654 ± 3.34%
348	100	0.1747 ± 0.19%	3.2202 ± 0.32%	0.0132 ± 1.49%	0.2829 ± 0.17%	3.6884 ± 0.36%	0.0463 ± 4.09%
	200	0.1617 ± 0.09%	1.7968 ± 0.18%	0.0100 ± 1.31%	0.2685 ± 0.15%	1.8498 ± 0.40%	0.0315 ± 2.77%
	300	0.1478 ± 0.14%	1.3962 ± 0.21%	0.0059 ± 2.20%	0.2664 ± 0.14%	1.1900 ± 0.47%	0.0292 ± 3.49%
	320	0.1466 ± 0.15%	1.3775 ± 0.28%	0.0056 ± 2.33%	0.2674 ± 0.11%	1.1059 ± 0.51%	0.0295 ± 2.05%
	348	0.1432 ± 0.22%	1.4131 ± 0.33%	0.0051 ± 2.69%	0.2681 ± 0.10%	0.9909 ± 0.43%	0.0307 ± 2.18%
	360	0.1426 ± 0.17%	1.4322 ± 0.22%	0.0047 ± 3.17%	0.2678 ± 0.10%	0.9883 ± 0.42%	0.0302 ± 2.49%
	400	0.1418 ± 0.10%	1.4201 ± 0.17%	0.0047 ± 1.43%	0.2648 ± 0.12%	1.0238 ± 0.50%	0.0248 ± 3.89%
	500	0.1399 ± 0.26%	1.4517 ± 0.35%	0.0043 ± 3.94%	0.2638 ± 0.17%	1.0248 ± 0.65%	0.0255 ± 5.65%
	600	0.1405 ± 0.16%	1.4392 ± 0.20%	0.0047 ± 2.87%	0.2650 ± 0.14%	0.9917 ± 0.74%	0.0245 ± 3.85%

P50052	50				0.3024 ± 0.85%	7.4294 ± 1.70%	0.0657 ± 6.19%
363	100	0.1795 ± 0.16%	3.1869 ± 0.26%	0.0132 ± 1.42%	0.2818 ± 0.25%	3.6993 ± 0.55%	0.0458 ± 3.44%
	200	0.1660 ± 0.18%	1.8701 ± 0.30%	0.0096 ± 1.98%	0.2698 ± 0.21%	1.8027 ± 0.58%	0.0341 ± 4.60%
	300	0.1550 ± 0.22%	1.3995 ± 0.36%	0.0066 ± 2.97%	0.2643 ± 0.14%	1.2232 ± 0.42%	0.0273 ± 3.55%
	330	0.1512 ± 0.12%	1.4130 ± 0.23%	0.0057 ± 1.30%	0.2654 ± 0.18%	1.0822 ± 0.68%	0.0274 ± 5.32%
	363	0.1509 ± 0.18%	1.3881 ± 0.27%	0.0057 ± 3.53%	0.2687 ± 0.24%	0.9676 ± 1.00%	0.0332 ± 7.75%
	380	0.1489 ± 0.12%	1.4138 ± 0.19%	0.0051 ± 1.17%	0.2651 ± 0.30%	0.9806 ± 1.28%	0.0270 ± 11.76%
	400	0.1474 ± 0.20%	1.4335 ± 0.32%	0.0048 ± 3.27%	0.2634 ± 0.15%	0.9773 ± 0.75%	0.0271 ± 11.41%
	500	0.1471 ± 0.08%	1.4350 ± 0.16%	0.0049 ± 1.13%	0.2613 ± 0.21%	0.9998 ± 1.05%	0.0226 ± 7.60%
	600	0.1457 ± 0.28%	1.4640 ± 0.54%	0.0046 ± 3.24%	0.2662 ± 0.15%	0.9498 ± 0.79%	0.0250 ± 7.76%

Q18179	50				0.3008 ± 0.70%	7.6673 ± 1.23%	0.0625 ± 5.34%
455	100	0.1798 ± 0.33%	3.7190 ± 0.59%	0.0103 ± 2.84%	0.2845 ± 0.16%	3.5814 ± 0.35%	0.0485 ± 2.86%
	200	0.1723 ± 0.16%	1.9839 ± 0.32%	0.0087 ± 1.50%	0.2685 ± 0.14%	1.8391 ± 0.49%	0.0302 ± 3.81%
	300	0.1609 ± 0.25%	1.4302 ± 0.40%	0.0059 ± 4.49%	0.2632 ± 0.16%	1.2382 ± 0.53%	0.0262 ± 4.69%
	420	0.1569 ± 0.27%	1.3665 ± 0.52%	0.0050 ± 2.90%	0.2636 ± 0.17%	0.8441 ± 0.59%	0.0222 ± 9.17%
	450	0.1590 ± 0.25%	1.3225 ± 0.61%	0.0052 ± 2.86%	0.2611 ± 0.13%	0.8203 ± 0.43%	0.0209 ± 4.93%
	455	0.1548 ± 0.26%	1.4038 ± 0.52%	0.0049 ± 2.76%	0.2655 ± 0.12%	0.7670 ± 0.49%	0.0246 ± 8.35%
	480	0.1557 ± 0.38%	1.3664 ± 0.67%	0.0051 ± 7.10%	0.2610 ± 0.10%	0.7929 ± 0.41%	0.0197 ± 6.70%
	500	0.1521 ± 0.45%	1.4145 ± 0.77%	0.0044 ± 5.30%	0.2615 ± 0.17%	0.7783 ± 0.62%	0.0204 5.09%
	600	0.1540 ± 0.25%	1.3886 ± 0.43%	0.0043 ± 3.72%	0.2596 ± 0.14%	0.7706 ± 0.60%	0.0174 ± 5.71%

P35348	50				0.3046 ± 0.61%	7.3443 ± 1.17%	0.0668 ± 4.85%
466	100	0.1809 ± 0.18%	3.1996 ± 0.28%	0.0135 ± 2.06%	0.2839 ± 0.22%	3.6314 ± 0.49%	0.0465 ± 2.49%
	200	0.1625 ± 0.12%	1.8687 ± 0.18%	0.0079 ± 1.63%	0.2696 ± 0.15%	1.8030 ± 0.48%	0.0315 ± 3.97%
	300	0.1643 ± 0.10%	1.2089 ± 0.15%	0.0086 ± 2.23%	0.2620 ± 0.13%	1.2472 ± 0.47%	0.0241 ± 5.52%
	400	0.1510 ± 0.24%	1.2641 ± 0.39%	0.0051 ± 2.76%			
	450	0.1521 ± 0.33%	1.2357 ± 0.55%	0.0050 ± 5.39%	0.2647 ± 0.16%	0.7874 ± 0.67%	0.0246 ± 3.93%
	466	0.1485 ± 0.17%	1.2982 ± 0.35%	0.0046 ± 2.93%			
	480	0.1517 ± 0.23%	1.2359 ± 0.34%	0.0056 ± 5.27%	0.2609 ± 0.25%	0.7981 ± 1.25%	0.0207 ± 9.36%
	500	0.1492 ± 0.22%	1.2845 ± 0.35%	0.0048 ± 3.64%	0.2668 ± 0.09%	0.7124 ± 0.49%	0.0265 ± 6.00%
	600	0.1509 ± 0.28%	1.2383 ± 0.40%	0.0050 ± 3.86%			

Fit parameters λ, λ_2 _and *K *of the modified Gumbel distribution for (FQPS) and (RQGS).

**Table 5:** Fit parameters for the HMM

		HMM n = 0			HMM n = 1		
***L***_**Q**_	***L***_**S**_	λ	10^4 ^λ_2_	10^3^*K*	λ	10^4 ^λ_2_	10^3^*K*
348	150	0.2890 ± 0.85%		49.4722 ± 7.27%	0.2310 ± 9.32%		21.4600 ± 66.56%
	200	0.2894 ± 2.84%		50.0796 ± 24.47%	0.2274 ± 1.74%		20.1017 ± 13.25%
	300	0.2895 ± 2.69%		53.3472 ± 24.00%	0.2240 ± 4.86%		17.8934 ± 37.22%
	348	0.2988 ± 3.24%		72.2356 ± 30.15%	0.2234 ± 2.39%		16.8704 ± 18.79%
	360	0.2895 ± 1.79%		51.9056 ± 16.04%	0.2220 ± 2.14%		16.3757 ±16.52%
	400	0.2859 ± 3.49%		48.4496 ± 31.10%	0.2232 ± 2.40%		17.5141 ± 18.94%
	500	0.2912 ± 6.63%		54.0687 ± 61.22%	0.2182 ± 2.39%		14.7371 ± 19.10%
	600	0.2901 ± 3.38%		51.9412 ± 31.74%	0.2180 ± 2.59%		14.2439 ± 20.86%

		HMM n = 2			HMM n = 3		
*L*_Q_	*L*_S_	λ	10^4 ^λ_2_	*K*	λ	10^4 ^λ_2_	*K*

348	150	0.1968 ± 0.70%	2.9247 ± 1.37%	12.0400 ± 6.48%	0.1767 ± 0.44%	2.6797 ± 1.01%	7.4435 ± 3.72%
	200	0.1947 ± 2.12%		9.8704 ± 14.29%	0.1795 ± 0.46%	2.3586 ± 0.92%	8.5733 ± 3.87%
	300	0.1937 ± 3.60%		9.9597 ± 25.32%	0.1863 ± 0.41%	2.0008 ± 0.94%	11.7859 ± 5.63%
	348	0.1888 ± 3.19%		8.1338 ± 22.42%	0.1876 ± 0.32%	1.9328 ± 0.89%	12.1223 ± 3.83%
	360	0.1926 ± 3.17%		9.7957 ± 22.82%	0.1853 ± 0.27%	1.9530 ± 0.65%	10.8640 ± 2.65%
	400	0.1934 ± 1.05%		9.9321 ± 8.22%	0.1757 ± 1.64%		7.1756 ± 11.58%
	500	0.1919 ± 1.61%		9.3630 ± 12.32%	0.1783 ± 0.98%		7.7945 ± 7.18%
	600	0.1912 ± 1.70%		9.3303 ± 13.25%	0.1768 ± 1.01%		7.4165 ± 8.19%

		HMM n = 4			HMM n = 5		
*L*_Q_	*L*_S_	λ	10^4 ^λ_2_	10^3^*K*	λ	10^4 ^λ_2_	10^3^*K*

348	150	0.1732 ± 0.47%	2.2119 ± 1.14%	7.4991 ± 6.08%	0.1710 ± 0.38%	2.0698 ± 0.92%	8.1950 ± 3.70%
	200	0.1686 ± 0.28%	2.1187 ± 0.72%	6.4162 ± 3.14%	0.1657 ± 0.39%	1.8231 ± 1.14%	6.9148 ± 3.82%
	300	0.1682 ± 0.36%	1.9635 ± 0.79%	6.5436 ± 4.22%	0.1599 ± 0.37%	1.7836 ± 0.79%	5.4451 ± 3.85%
	348	0.1685 ± 0.35%	1.9408 ± 0.74%	7.3851 ± 3.34%	0.1580 ± 0.28%	1.7930 ± 0.68%	5.3049 ± 2.61%
	360	0.1678 ± 0.42%	1.9421 ± 0.92%	6.5775 ± 4.07%	0.1605 ± 0.23%	1.7481 ± 0.50%	5.7512 ± 2.89%
	400	0.1662 ± 0.18%	1.9782 ± 0.40%	6.4164 ± 2.32%	0.1587 ± 0.28%	1.7828 ± 0.73%	5.4513 ± 2.57%
	500	0.1693 ± 0.24%	1.9047 ± 0.51%	7.0735 ± 2.11%	0.1587 ± 0.16%	1.7957 ± 0.40%	5.4770 ± 2.31%
	600	0.1693 ± 0.17%	1.8994 ± 0.39%	7.1112 ± 2.06%	0.1575 ± 0.29%	1.8330 ± 0.58%	5.2125 ± 2.68%

		HMM n = 6			HMM n = 7		
*L*_Q_	*L*_S_	λ	10^4 ^λ_2_	10^3^*K*		10^4 ^λ_2_	10^3^*K*

348	150	0.1663 ± 0.49%	2.1403 ± 1.04%	7.9392 ± 5.83%	0.1646 ± 0.30%	2.1396 ± 0.65%	8.7088 ± 4.21%
	200	0.1614 ± 0.25%	1.7767 ± 0.65%	6.7568 ± 2.30%	0.1574 ± 0.41%	1.7687 ± 1.17%	6.5219 ± 3.81%
	300	0.1551 ± 0.28%	1.5986 ± 0.80%	5.2551 ± 3.18%	0.1514 ± 0.26%	1.4638 ± 0.62%	5.0238 ± 4.34%
	348	0.1531 ± 0.20%	1.5993 ± 0.55%	4.9132 ± 2.71%	0.1482 ± 0.33%	1.4755 ± 0.77%	4.4535 ± 4.13%
	360	0.1536 ± 0.34%	1.6036 ± 1.02%	4.9160 ± 3.41%	0.1490 ± 0.39%	1.4479 ± 0.93%	4.6858 ± 3.28%
	400	0.1537 ± 0.27%	1.5713 ± 0.62%	4.9524 ± 3.05%	0.1494 ± 0.24%	1.4328 ± 0.70%	4.6867 ± 2.08%
	500	0.1519 ± 0.23%	1.6229 ± 0.67%	4.6812 ± 2.14%	0.1472 ± 0.29%	1.4706 ± 0.63%	4.2881 ± 2.50%
	600	0.1489 ± 0.15%	1.7148 ± 0.33%	4.2283 ± 2.16%	0.1460 ± 0.18%	1.5193 ± 0.49%	4.2679 ± 1.74%

		HMM n = 8			HMM n = 9		
*L*_Q_	*L*_S_	λ	10^4 ^λ_2_	10^3^*K*	λ	10^4 ^λ_2_	10^3^*K*

348	150	0.1595 ± 0.47%	2.2162 ± 1.01%	7.5355 ± 4.01%	0.1603 ± 0.23%	2.1517 ± 0.48%	8.0273 ± 2.17%
	200	0.1534 ± 0.55%	1.8019 ± 1.46%	5.9224 ± 5.25%	0.1508 ± 0.14%	1.7854 ± 0.28%	6.3535 ± 1.89%
	300	0.1473 ± 0.47%	1.3916 ± 1.24%	4.8483 ± 4.01%	0.1413 ± 0.12%	1.4118 ± 0.35%	4.2141 ± 1.43%
	348	0.1458 ± 0.32%	1.3409 ± 0.85%	4.6141 ± 3.69%	0.1398 ± 0.10%	1.3281 ± 0.33%	3.9661 ± 1.44%
	360	0.1469 ± 0.34%	1.2868 ± 0.90%	4.9271 ± 2.73%	0.1400 ± 0.16%	1.2888 ± 0.43%	4.0126 ± 1.79%
	400	0.1440 ± 0.34%	1.3591 ± 1.05%	4.0064 ± 3.48%	0.1382 ± 0.25%	1.2954 ± 0.67%	3.7257 ± 2.14%
	500	0.1433 ± 0.29%	1.3382 ± 0.85%	3.9952 ± 2.70%	0.1352 ± 0.14%	1.3472 ± 0.42%	3.1780 ± 1.68%
	600	0.1416 ± 0.33%	1.3760 ± 0.94%	3.7782 ± 3.14%	0.1359 ± 0.13%	1.3399 ± 0.38%	3.3536 ± 1.49%

		HMM n = 10			HMM n = 11		
*L*_Q_	*L*_S_	λ	10^4 ^λ_2_	10^3^*K*	λ	10^4 ^λ_2_	10^3^*K*

348	150	0.1552 ± 0.14%	2.2225 ± 0.30%	6.7936 ± 2.08%	0.1455 ± 0.14%	2.3813 ± 0.15%	4.9660 ± 3.82%
	200	0.1459 ± 0.22%	1.8336 ± 0.37%	5.7585 ± 3.30%	0.1417 ± 0.17%	1.8428 ± 0.35%	5.1264 ± 2.07%
	300	0.1370 ± 0.22%	1.4024 ± 0.56%	3.8087 ± 1.79%	0.1324 ± 0.27%	1.3842 ± 0.68%	3.2129 ± 2.79%
	348	0.1353 ± 0.15%	1.2962 ± 0.38%	3.5507 ± 1.68%	0.1316 ± 0.22%	1.2518 ± 0.69%	3.1546 ± 1.94%
	360	0.1343 ± 0.13%	1.2830 ± 0.36%	3.4674 ± 1.39%	0.1297 ± 0.25%	1.2737 ± 0.52%	2.9445 ± 2.81%
	400	0.1334 ± 0.16%	1.2602 ± 0.38%	3.2164 ± 1.71%	0.1302 ± 0.20%	1.2160 ± 0.56%	2.9704 ± 1.59%
	500	0.1307 ± 0.16%	1.3013 ± 0.46%	2.8331 ± 1.22%	0.1280 ± 0.30%	1.2426 ± 0.86%	2.7433 ± 2.73%
	600	0.1305 ± 0.23%	1.3097 ± 0.56%	2.8239 ± 1.82%	0.1257 ± 0.22%	1.2908 ± 0.55%	2.4921 ± 1.79%

The table shows the fit parameters of the score distribution Prob(*S *= *s*| # of helices = n) for 1 ≤ *n *≤ 11 for *L_Q _*= 348 and different subject lengths. For entries, where λ_2 _is left out, a suitable fit (with a small reduced χ^2 ^value) to the modified Gumbel distribution Eq. (15) was not possible and only the Gumbel parameters of the high probability region are shown.
